# A Preoperative Scoring System to Predict the Risk of Inadequate Lymph Node Count in Rectal Cancer

**DOI:** 10.3389/fonc.2022.938996

**Published:** 2022-07-08

**Authors:** Hao Zhang, Chunlin Wang, Yunxiao Liu, Hanqing Hu, Guiyu Wang

**Affiliations:** Department of Colorectal Surgery, the Second Affiliated Hospital of Harbin Medical University, Harbin, China

**Keywords:** rectal cancer, lymph node examination, preoperative factor, scoring system, risk model

## Abstract

**Purpose:**

The aim of this study was to develop and validate a preoperative scoring system to stratify rectal cancer (RC) patients with different risks of inadequate lymph node examination.

**Methods:**

A total of 1,375 stage I–III RC patients between 2011 and 2020 from the Second Affiliated Hospital of Harbin Medical University were included in the retrospective study and randomly divided into a development set (*n* = 688) and a validation set (*n* = 687). The logistic regression model was used to determine independent factors contributing to lymph node count (LNC) < 12. A preoperative scoring system was constructed based on beta (*β*) coefficients. The area under the receiver operating curve (AUC) was used to test model discrimination.

**Results:**

Preoperative significant indicators related to LNC < 12 included age, tumor size, tumor location, and CEA. The AUCs of the scoring system for development and validation sets were 0.694 (95% CI = 0.648–0.741) and 0.666 (95% CI = 0.615–0.716), respectively. Patients who scored 0–2, 3–4, and 5–6 were classified into the low-risk group, medium-risk group, and high-risk group, respectively.

**Conclusions:**

The preoperative scoring system could identify RC patients with high risk of inadequate lymphadenectomy accurately and further provide a reference to perform preoperative lymph node staining in targeted patients to reduce the difficulty of meeting the 12-node standard, with the purpose of accurate tumor stage and favorable prognosis.

## Introduction

Globally, rectal cancer (RC) poses a major public health challenge due to its high incidence and mortality rate (1). In the treatment of RC patients, lymph node count (LNC), which is the pivotal determinant to guarantee the accuracy of pathological stage and essential for the selection of appropriate therapeutic strategies subsequently, is deemed as a vital pathological parameter obtained from the surgical specimen routinely (2). Moreover, it is also a datum that could identify the performance of radical surgery objectively. Hence, adequate LNC is usually required in clinical practice.

The American Society of Clinical Oncology (ASCO) and the National Comprehensive Cancer Network (NCCN) guidelines advocate that at least 12 lymph nodes should be harvested to ensure proper lymphadenectomy and accurate tumor stage. Patients with inadequate lymphadenectomy (LNC < 12) are characterized by a higher probability of stage migration, leading to the error of the subsequent therapeutic method (3). However, LNC is a completely postoperative parameter and has been generally influenced by anatomical features, tumor characteristics, patient-related factors, and technical factors (surgical and anatomopathological factors) (4–7).

Nowadays, lymphatic tracers such as carbon nanoparticle suspension and methylene blue have been gradually applied in clinical practice to make the lymph node more visible to surgeons and pathologists and improve the detection and biopsy rates of lymph nodes (8, 9). However, the cost might limit their application to some extent. With these premises, using preoperative information to assess the risk of inadequate lymphadenectomy foreseeingly and performing targeted application of lymph node staining for patients with high risk of inadequate lymph node examination would be of eminent significance.

In recent years, the risk prediction model has been applied widely to help clinicians determine the risk of an outcome event. For RC patients, many models have been built to predict the postoperative prognosis, therapeutic effects, and likelihood of lymph node metastasis (10–12). A risk model of predicting LNC < 12 could be a helpful method to recognize an individual’s risk level of inadequate lymph node examination. However, a relevant prediction model to determine the risk of inadequate lymphadenectomy in RC is still lacking. Therefore, the goal of the current study was to construct a scoring system to identify RC patients with high risk of inadequate lymphadenectomy promptly using preoperative factors.

## Methods

### Study Population

Rectal cancer patients from the Second Affiliated Hospital of Harbin Medical University between January 2011 and December 2020 were enrolled in the retrospective study. Inclusion criteria included the following: (1) radical resection was the first course of treatment; (2) aged ≥ 18 years; (3) patients diagnosed as stage I–III RC pathologically; (4) RC was the only malignancy; and (5) histological types were limited to adenocarcinoma and mucous/signet-ring cell carcinoma. Exclusion criteria included the following: (1) patients underwent neoadjuvant therapy and (2) patients with unknown LNC, age, gender, BMI, tumor location, tumor size, and CEA. Candidate predictors included age (<60, 60–74, and ≥75 years old), gender (male and female), BMI (<25 and ≥25 kg/m^2^), tumor size (<5 and ≥5 cm), tumor location (upper rectum, middle rectum, and lower rectum) and CEA (<5 and ≥5 ng/ml).

In this study, standard surgical procedures including the ligation of the inferior mesenteric artery and total mesorectal excision (TME) principle were performed routinely. The pathologist applied manual technique including serial sections, inspection, and palpation in routine gross specimen handling. Furthermore, specimens with LNE < 12 were reexamined by the pathologist and a fat clearance technique using Carnoy’s solution was performed to increase the yield.

### Statistical Analysis

All patients were divided into a development set and a validation set with a 1:1 ratio randomly. The characteristics of the development set and validation set were compared using Pearson’s chi-squared test. Based on cancer-specific survival (CSS), Kaplan–Meier curves were drawn and data were analyzed using the log-rank test. The logistic regression model was used to determine independent factors contributing to LNC < 12. Potential indicators with *p* < 0.20 in the univariate analysis were introduced into the multivariate analysis. Risk predictors with *p* < 0.05 were used to construct the scoring system. The discrimination of the scoring system was appraised by the area under the receiver operating curve (AUC). The predicted rate of inadequate lymphadenectomy was calculated from the model regression formula. Moreover, the comparison between predicted (mean ± SD) and observed rates of LNC < 12 was used to test the calibration and the model fit was further evaluated by the Hosmer–Lemeshow (H–L) test. *p* < 0.05 (two-sided) was defined as statistically significant. All statistical analyses were performed using SPSS 22.0.

### Scoring Method

Each predictor in the multivariate model was associated with a beta (*β*) coefficient. The score of each significant predictor was achieved by dividing the *β* coefficient through the lowest *β* coefficient in the logistic regression model and rounding to the nearest integer (5). The individual score was calculated by summing the score of each factor.

## Results

### Patient Characteristics

In this retrospective study, a total of 1,375 RC patients were included, of whom 262 (19.1%) patients had inadequate lymph node examination. After random division, 688 patients (133 patients with LNC < 12, 19.3%) were in the development set and 687 patients (129 patients with LNC < 12, 18.8%) were in the validation set. Furthermore, Pearson’s chi-squared test indicated that no significant difference could be found in age (*p* = 0.343), gender (*p* = 0.087), BMI (*p* = 0.755), tumor size (*p* = 0.556), tumor location (*p* = 0.487), CEA (*p* = 0.185), and LNC (*p* = 0.794) between the two sets (**Table 1**).

**Table 1:** Characteristics of patients in the development and validation sets.

Characteristics		Development set (*N* = 688)	Validation set (*N* = 687)	*p*
**Age (years), *n* (%)**	<60	289 (42.0)	298 (43.4)	0.343
	60–74	334 (48.5)	311 (45.3)	
	≥75	65 (9.5)	78 (11.3)	
**Gender, *n* (%)**	Male	460 (62.4)	429 (66.9)	0.087
	Female	228 (37.6)	258 (33.1)	
**BMI (kg/m^2^), *n* (%)**	<25	484 (70.3)	478 (69.6)	0.755
	≥25	204 (29.7)	209 (30.4)	
**Tumor size (cm), *n* (%)**	≥5	269 (39.1)	258 (37.6)	0.556
	<5	419 (60.9)	429 (62.4)	
**Tumor location, *n* (%)**	Upper (≥10 cm)	204 (29.7)	209 (30.4)	0.487
	Middle (5–10 cm)	255 (37.1)	234 (34.1)	
	Lower (<5 cm)	229 (33.2)	244 (35.5)	
**CEA (ng/ml), *n* (%)**	≥5	262 (38.1)	238 (34.6)	0.185
	<5	426 (61.9)	449 (65.4)	
**LNC, *n* (%)**	<12	133 (19.3)	129 (18.8)	0.794
	≥12	555 (80.7)	558 (81.2)	

### Survival Analysis

**Figure 1:**
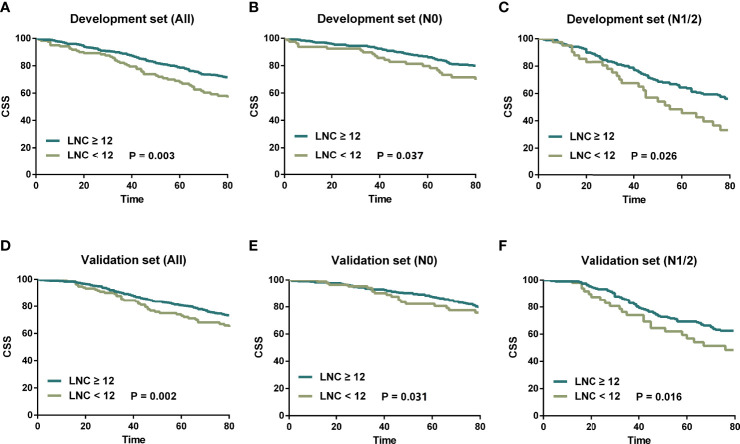
Kaplan–Meier survival curves stratified by LNC (≥12 vs. <12). **(A)** LNC ≥ 12 vs. LNC < 12 in the development set. **(B)** LNC ≥ 12 vs. LNC < 12 for N0 patients in the development set. **(C)** LNC ≥ 12 vs. LNC < 12 for N1/2 patients in the development set. **(D)** LNC ≥ 12 vs. LNC < 12 in the validation set. **(E)** LNC ≥ 12 vs. LNC < 12 for N0 patients in the validation set. **(F)** LNC ≥ 12 vs. LNC < 12 for N1/2 patients in the validation set.

To begin with, all patients were classified into two subsets (LNC ≥ 12 and LNC < 12) to determine the prognostic impact of the number of lymph node examination. According to the CSS, Kaplan–Meier survival curves are plotted in **Figure 1** and we revealed that in both the development set and validation set, patients with LNC ≥ 12 posed better CSS compared to those with LNC < 12 (*p* = 0.003 for the development set, *p* = 0.002 for the validation set). Moreover, the favorable CSS of LNC ≥ 12 could also be found in N0 (*p* = 0.037 for the development set, *p* = 0.031 for the validation set) and N1/2 (*p* = 0.026 for the development set, *p* = 0.016 for the validation set) patients in both development and validation sets.

### Risk Factors for LNC < 12 in the Development Set

**Figure 2:**
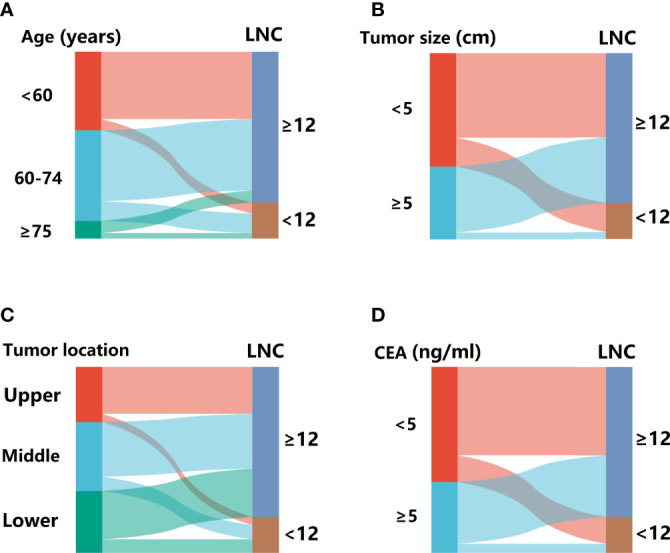
The Sankey diagram for the proportion of patients with LNC < 12 in different subsets. **(A)** Stratified by age. **(B)** Stratified by tumor size. **(C)** Stratified by tumor location. **(D)** Stratified by CEA.

Univariate analysis revealed the association between each factor and inadequate lymphadenectomy, including age (*p* = 0.004), gender (*p* = 0.825), BMI (*p* = 0.166), tumor size (*p* < 0.001), tumor location (*p* = 0.134), and CEA (*p* = 0.001) (**Table 2**). Then, potential indicators with *p* < 0.20 including age, BMI, tumor size, tumor location, and CEA were used to perform the multivariate logistic model. Multivariate analysis indicated that age (OR = 1.803, 95% CI = 1.165–2.788 for aged 60–74 years, *p* = 0.008; OR = 2.978, 95% CI = 1.555–5.703 for aged ≥75 years, *p* = 0.001; using aged <60 years as the reference), tumor size (OR = 3.092, 95% CI = 1.926–4.964 for tumor size < 5 cm, *p* < 0.001; using tumor size ≥ 5 cm as the reference), tumor location (OR = 1.771, 95% CI = 1.052–2.982 for middle rectum, *p* = 0.021; OR = 1.704, 95% CI = 1.021–2.844 for lower rectum, *p* = 0.031; using upper rectum as the reference), and CEA level (OR = 1.858, 95% CI = 1.194–2.892 for CEA < 5 ng/ml, *p* = 0.006; using CEA ≥ 5 ng/ml as the reference) were still the statistically significant predictors related to LNC < 12 (**Table 3**). In addition, the Sankey diagram vividly showed the different proportions of LNC < 12 in patients stratified by age, tumor size, tumor location, and CEA level (**Figures 2A–D**).

**Table 2:** Univariate logistic regression analysis in the development set.

Characteristics		Number	OR [95% CI]	*p*
**Age (years)**	<60	289	1	0.004
	60-74	334	1.662 [1.091–2.532]	
	≥75	65	2.688 [1.444–5.006]	
**Gender**	Male	460	1	0.825
	Female	228	0.956 [0.638–1.431]	
**BMI (kg/m^2^)**	<25	484	1	0.166
	≥25	204	0.753 [0.504–1.125]	
**Tumor size (cm)**	≥5	269	1	<0.001
	<5	419	3.205 [2.023–5.078]	
**Tumor location**	Upper (≥10 cm)	204	1	0.134
	Middle (5–10 cm)	255	1.620 [0.984–2.667]	
	Lower (<5 cm)	229	1.522 [0.931–2.488]	
**CEA (ng/ml)**	≥5	262	1	0.001
	<5	426	2.030 [1.328–3.105]	

**Table 3:** Multivariate logistic regression analysis in the development set.

Characteristics		OR [95% CI]	*p*
**Age (years)**	<60	1	
	60–74	1.803 [1.165–2.788]	0.008
	≥75	2.978 [1.555–5.703]	0.001
**Tumor size (cm)**	≥5	1	
	<5	3.092 [1.926–4.964]	<0.001
**Tumor location**	Upper (≥10 cm)	1	
	Middle (5–10 cm)	1.771 [1.052–2.982]	0.021
	Lower (<5 cm)	1.704 [1.021–2.844]	0.031
**CEA (ng/ml)**	≥5	1	
	<5	1.858 [1.194–2.892]	0.006

### Risk Scores

According to the *β* coefficient in the logistic regression model, each predictor was linked to a score as previously described. The detailed scores for each predictor are shown in **Table 4**. The total scores of patients were calculated by adding up the scores of each item. In this study, the lowest score was 0 point and the highest score was 6 points.

**Table 4:** Scoring system.

Characteristics		*β* coefficient	Score
**Age (years)**	<60	**-**	–
	60–74	0.589	1
	≥75	1.091	2
**Tumor size (cm)**	≥5	**-**	–
	<5	1.129	2
**Tumor location**	Upper (≥10 cm)	**-**	–
	Middle (5–10 cm)	0.572	1
	Lower (<5 cm)	0.533*	1
**CEA (ng/ml)**	≥5	**-**	–
	<5	0.620	1

*The lowest β coefficient.

### Model Accuracy and Validity

**Figure 3:**
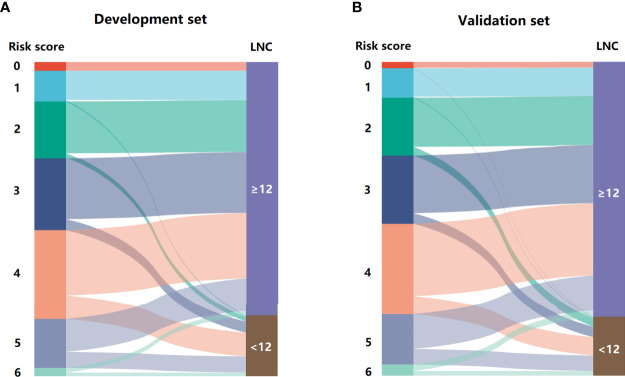
The Sankey diagram for the proportion of LNC < 12 in patients with different risk scores. **(A)** development set and **(B)** validation set.

**Figure 4:**
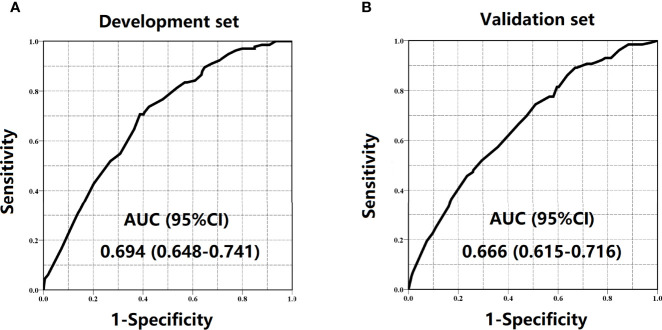
ROC curves in the **(A)** development set and **(B)** validation set.

**Figure 5:**
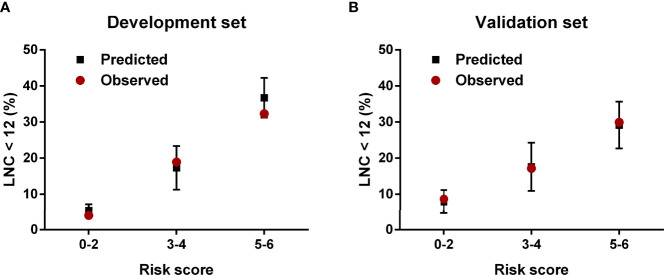
The predicted and observed rates of LNC < 12 in the **(A)** development set and **(B)** validation set.

The regression analysis indicated that an increase in 1 score was associated with the increased risk of inadequate lymph node examination by 1.763 times (95% CI = 1.494–2.080) for the development set and 1.513 times (95% CI = 1.296–1.767) for the validation set. As shown in **Figure 3**, the proportion of patients with LNC < 12 showed an increasing trend with the risk score varying from 0 point to 6 points in both development and validation sets. The AUCs for development and validation sets were 0.694 (95% CI = 0.648–0.741) and 0.666 (95% CI = 0.615–0.716) (**Figure 4**), respectively, exhibiting a favorable discrimination of the model to accurately predict the risk of inadequate lymph node examination for RC patients. The H–L test for development (*p* = 0.620, *χ* ^2^ = 6.239) and validation sets (*p* = 0.498, *χ* ^2^ = 7.367) suggested that the model fitted well. Subsequently, risk classification was constructed based on total scores. Patients scored 0–2, 3–4, and 5–6 were stratified into the low-risk group, medium-risk group, and high-risk group, respectively. Then, the predicted rates (mean ± SD) and observed rates of LNC < 12 were compared in the three risk groups. As was shown in **Figure 5**, the predicted rates were in accord with the observed rates in both the development set (low-risk group: 5.47 ± 1.73 vs. 3.96; medium-risk group: 17.32 ± 6.03 vs. 18.94; high-risk group: 36.72 ± 5.49 vs. 32.33) and the validation set (low-risk group: 7.90 ± 3.26 vs. 8.70; medium-risk group: 17.60 ± 6.67 vs. 17.19; high-risk group: 29.16 ± 6.48 vs. 29.93).

## Discussion

In the treatment of RC patients, LNC presents a crucial role in evaluating postoperative pathological tumor staging accurately and is important for the rational selection of subsequent therapeutic strategies. The ASCO and NCCN recommend that at least 12 lymph nodes should be examined to guarantee a reliable postoperative pathological staging. However, LNC depends, to a large extent, on numerous variables, including patient-specific, tumor-related, hospital-dependent, and technique-varying factors (4–7). There are still some RC patients who underwent inadequate lymphadenectomy in clinical practice based on exhaustive population studies (13). Although preoperative factors could not be altered, preoperative information could help us assess the risk level of patients having inadequate lymph node examination, and subsequently, lymphatic tracers such as carbon nanoparticle suspension and methylene blue could be used to perform lymph node staining in high-risk patients to make lymph nodes more visible, therefore reducing the difficulty of meeting the 12-node standard. Hence, recognizing an individual’s risk level of inadequate lymph node examination before surgery seems to be crucial in current clinical practice.

In the study, the rate of inadequate lymph node examination was 19.1%, and it was lower than previous studies constructed by Fritzmann et al. (23.8%) and Orsenigo et al. (37.0%) (14, 15), which was probably because patients who received neoadjuvant therapy were excluded in this article, leading to a higher number of lymph node detections. Age, gender, BMI, tumor size, and tumor location are common easy-to-get preoperative parameters for RC patients. Moreover, the preoperative serum tumor marker CEA is also applied to evaluate surgically treated patients routinely. Therefore, these six preoperative factors were analyzed retrospectively in this work. The logistic regression model indicated that age, tumor size, tumor location, and CEA were significant factors associated with inadequate lymphadenectomy in RC. To our knowledge, the reasons leading to these results were multi-factorial. Elderly patients have been stated in many studies to be related to a notable reduction in the number of lymph nodes retrieved. The probable reason was that due to the poor physical tolerance of elderly patients, the surgery for them was relatively conservative, and therefore, young patients might receive a more extensive lymphadenectomy compared with elderly patients, resulting in the phenomenon that increasing age was associated with higher risk of LNC < 12. Furthermore, some scholars stated that a lymphatic involution would occur with age, which might reduce the nodal size and make them harder to be detected (16–18). In this study, tumor size was another predictor in the proposed scoring system. Nash et al. revealed that a more robust inflammatory and lymphocytic antitumor response could be found in patients with larger tumors (19). Furthermore, patients with large lesions were more likely to require an extended surgical resection, therefore increasing the length of the specimen, a parameter positively impacting the number of lymph nodes retrieved (20). Consistent with previous works, patients with higher RC had less risk of inadequate lymphadenectomy compared with those having middle and lower rectal tumors (13, 21), although the potential mechanism resulting in the phenomenon was still unclear. To the best of our knowledge, as a result of the anatomical features, resected higher RC specimens often contain a larger amount of mesentery compared with middle and lower RC specimens, which might be the reason for the increasing number of lymph nodes examined in higher RC patients. There was also a negative association between CEA level and LNC. Patients with CEA-positive diseases were usually characterized by more aggressive histologic features and advanced tumors, which were accompanied by prominent lymphocytic antitumor reaction and a higher probability of lymph node metastasis (22–24), making lymph nodes more visible to pathologists and surgeons and reducing the difficulty of meeting the 12-node measure.

Subsequently, we assessed the weight of each indicator and constructed a scoring system (range 0–6 points) to stratify RC patients with a different risk level of inadequate lymph node examination. The AUCs of the proposed scoring system for the development set and the validation set were 0.694 (95% CI = 0.648–0.741) and 0.666 (95% CI = 0.615–0.716), respectively. The H–L test for both sets also demonstrated that the model had no significant lack of fit. Moreover, patients were stratified into low-risk (0–2 points), medium-risk (3–4 points), and high-risk (5–6 points) groups based on their scores. The calibration posed a satisfying consistency between observed and predicted rates of LNC < 12 in development and validation sets. The findings of the research present significant implications in clinical practice. According to the preoperative scoring system, we could easily determine these RC patients with a high risk of inadequate lymph node examination before surgery and perform targeted lymph node staining. However, this tool could only provide a potential reference, but not guidance. In the decision-making process, clinicians should still take the diligence of pathologists, the performance of surgeons, and the actual condition of patients into consideration, and not just depend on the scoring system alone.

Neoadjuvant therapy, as a crucial part in the treatment of RC patients, has been found to be associated with a significantly reduced LNC in surgical specimens. There were some studies exploring the minimum LNC for RC patients undergoing preoperative radiochemotherapy, and whether the 12-node standard should be applied to those with neoadjuvant treatment is still under debate (25, 26). Up to now, there is no consensus on the optimal minimum LNC for this special population. Therefore, although neoadjuvant treatment poses a vital role in RC, patients with preoperative therapy were not included in the study, the aim of which was to predict the likelihood of LNC < 12, and the further explosion on the targeted nomogram for this population is worthy of encouragement. Moreover, unresectable recurrent RC patients progressing after systemic chemotherapy and radiotherapy were also a special population that needs to be taken into account. The therapy for these patients required a multidisciplinary therapeutic scheme, and when standard treatments such as systemic radiochemotherapy were impracticable, the combination of systemic therapy and a locoregional chemotherapy could be an alternative method (27). Hence, a targeting model to predict clinical benefit for them should also be constructed.

Preoperative imaging poses an important role in RC patients. However, the accuracy of preoperative MRI and CT is not stable as we expected (28). On the one hand, preoperative T staging of rectal tumor is a difficult task for radiologists and over-staging often occurs due to the perirectal desmoplastic reaction that does not contain tumor cells. On the other hand, the accuracy of preoperative staging also largely depends on the experience of radiologists. Although preoperative staging is a crucial parameter for clinicians, its accuracy could not be guaranteed in some cases. A wrong assessment of preoperative staging might result in a completely different risk level of inadequate lymph node examination and influence clinicians to make a reasonable decision in practice. Furthermore, because the level of radiologists could not be guaranteed in some hospitals, the application of preoperative staging in the scoring system might limit its promotion to a large extent. Therefore, the preoperative TNM stage has not been included in the scoring system.

Since LNC is crucial for colorectal cancer patients, relevant studies for indicators impacting the extent of lymphadenectomy have been explored globally. Compared to other studies, the merits of this article were twofold. On the one hand, it was the first study to weigh each indicator’s contribution to LNC < 12 and propose an easy-to-use scoring manual to preoperatively predict the risk of inadequate LNC for RC patients. On the other hand, the model consists of accessible tumor and clinical parameters, suggesting that it could be acceptable clinically and present eminent significance in clinical work.

As another parameter utilized to assess lymph nodal stages, lymph node ratio, which was defined as positive LNC divided by the total LNC, could partly overcome the dependence of nodal stage on the extent of lymphadenectomy, and it has been considered as a robust prognostic indicator in many papers (29, 30). Therefore, in the field of lymph node status assessment, besides LNC, lymph node ratio is another nodal datum worthy of exploring.

Still, the authors acknowledged several study limitations. Firstly, the data were collected and processed retrospectively and it was inevitable to have observer and confusion bias. Secondly, indicators in the study were limited and incorporation of some novel factors might make the results more accurate and powerful. Thirdly, although the scoring system performed well in development and validation sets, it still needed to be verified by some external clinical works. Fourthly, due to the follow-up limitation of our own department, there are still some patients without survival data and we could not further perform a prognostic analysis in this cohort.

## Conclusion

In conclusion, the current study revealed that age, tumor size, tumor location, and CEA were significant preoperative factors in meeting the 12-node standard. Moreover, we developed and validated a scoring system to stratify RC patients with different risk levels of inadequate lymph node examination (LNC < 12) before surgery, which could provide a reference to perform preoperative lymph node staining in targeted patients to facilitate the harvest of lymph nodes after surgery and reduce the difficulty of meeting the 12-node standard.

## Data Availability Statement

The raw data supporting the conclusions of this article will be made available by the authors, without undue reservation.

## Ethics Statement

This study was approved by the Second Affiliated Hospital of Harbin Medical University. The study adhered to World Medical Association’s Declaration of Helsinki for Ethical Human Research. The informed consent was not required due to the retrospective nature of the study.

## Author Contributions

HZ and GW designed the study. HZ, CW, and YL collected data. HZ, HH, and GW analyzed and interpreted data. HZ and GW drafted the manuscript. HZ and GW critically revised the manuscript. All authors read and approved the final manuscript.

## Conflict of Interest

The authors declare that the research was conducted in the absence of any commercial or financial relationships that could be construed as a potential conflict of interest.

## Publisher’s Note

All claims expressed in this article are solely those of the authors and do not necessarily represent those of their affiliated organizations, or those of the publisher, the editors and the reviewers. Any product that may be evaluated in this article, or claim that may be made by its manufacturer, is not guaranteed or endorsed by the publisher.

## Abbreviations

RC, rectal cancer; LNC, lymph node count; ASCO, American Society of Clinical Oncology; NCCN, National Comprehensive Cancer Network; AUC, area under the receiver operating characteristic curve; CI, confidence intervals.

## References

[1] Siegel R Miller K Goding Sauer A Fedewa S Butterly L Anderson J. Colorectal Cancer Statistics, 2020. CA: Cancer J Clin (2020) 70(3):145–64. doi: 10.3322/caac.21601 32133645

[2] Amin MB Edge SB Greene FL. AJCC Cancer Staging Manual. 8th edition. Chicago, USA: Springer (2017).

[3] Compton C Greene F. The Staging of Colorectal Cancer: 2004 and Beyond. CA: Cancer J Clin (2004) 54(6):295–308. doi: 10.3322/canjclin.54.6.295 15537574

[4] Polignano F Henderson N Alishahi S Zito A. Laparoscopic Colectomy for Cancer and Adequate Lymphadenectomy: Association Between Survival and Number of Lymph Nodes. Surg Endoscopy (2006) 20(6):996–7. doi: 10.1007/s00464-005-0555-1 16739001

[5] Wang Y Guan X Zhang Y Zhao Z Gao Z Chen H. A Preoperative Risk Prediction Model for Lymph Node Examination of Stage I-III Colon Cancer Patients: A Population-Based Study. J Cancer (2020) 11(11):3303–9. doi: 10.7150/jca.41056 PMC7097944 32231735

[6] Dorrance H Docherty G O'Dwyer P. Effect of Surgeon Specialty Interest on Patient Outcome After Potentially Curative Colorectal Cancer Surgery. Dis Colon Rectum (2000) 43(4):492–8. doi: 10.1007/BF02237192 10789744

[7] Guan X Wang Y Hu H Zhao Z Jiang Z Liu Z. Reconsideration of the Optimal Minimum Lymph Node Count for Young Colon Cancer Patients: A Population-Based Study. BMC Cancer (2018) 18(1):623. doi: 10.1186/s12885-018-4428-0 29859052 PMC5984774

[8] Cai H He H Tian W Zhou M Hu Y Deng Y. Colorectal Cancer Lymph Node Staining by Activated Carbon Nanoparticles Suspension In Vivo or Methylene Blue In Vitro. World J Gastroenterol (2012) 18(42):6148–54. doi: 10.3748/wjg.v18.i42.6148 PMC3496893 23155345

[9] Tang L Sun L Zhao P Kong D. Effect of Activated Carbon Nanoparticles on Lymph Node Harvest in Patients With Colorectal Cancer. Colorectal Dis (2019) 21(4):427–31. doi: 10.1111/codi.14538 30580490

[10] Valentini V van Stiphout R Lammering G Gambacorta M Barba M Bebenek M. Nomograms for Predicting Local Recurrence, Distant Metastases, and Overall Survival for Patients With Locally Advanced Rectal Cancer on the Basis of European Randomized Clinical Trials. J Clin Oncol (2011) 29(23):3163–72. doi: 10.1200/JCO.2010.33.1595 21747092

[11] Kwon T Choi S Lee Y Kim J Oh S Lee I. Novel Methods of Lymph Node Evaluation for Predicting the Prognosis of Colorectal Cancer Patients With Inadequate Lymph Node Harvest. Cancer Res Treat (2016) 48(1):216–24. doi: 10.4143/crt.2014.312 PMC4720064 25943323

[12] Guan X Ma C Quan J Zhao Z Chen H Sun P. A Prognostic Index Model to Individually Predict Clinical Outcomes for Colorectal Cancer With Synchronous Bone Metastasis. J Cancer (2020) 11(15):4366–72. doi: 10.7150/jca.40921 PMC7255384 32489455

[13] Ahmadi O Stringer M Black M McCall J. Clinico-Pathological Factors Influencing Lymph Node Yield in Colorectal Cancer and Impact on Survival: Analysis of New Zealand Cancer Registry Data. J Surg Oncol (2015) 111(4):451–8. doi: 10.1002/jso.23848 25663298

[14] Fritzmann J Contin P Reissfelder C Büchler M Weitz J Rahbari N. Comparison of Three Classifications for Lymph Node Evaluation in Patients Undergoing Total Mesorectal Excision for Rectal Cancer. Langenbeck's Arch Surg (2018) 403(4):451–62. doi: 10.1007/s00423-018-1662-5 29523953

[15] Orsenigo E Gasparini G Carlucci M. Clinicopathological Factors Influencing Lymph Node Yield in Colorectal Cancer: A Retrospective Study. Gastroenterol Res Practice 2019 (2019), 5197914. doi: 10.1155/2019/5197914 PMC6362492 30804995

[16] Wang L Hollenbeak C Stewart D. Node Yield and Node Involvement in Young Colon Cancer Patients: Is There a Difference in Cancer Survival Based on Age? J gastrointestinal Surg (2010) 14(9):1355–61. doi: 10.1007/s11605-010-1275-y 20585992

[17] Tekkis P Smith J Heriot A Darzi A Thompson M Stamatakis J. A National Study on Lymph Node Retrieval in Resectional Surgery for Colorectal Cancer. Dis Colon Rectum (2006) 49(11):1673–83. doi: 10.1007/s10350-006-0691-2 17019656

[18] Morris E Maughan N Forman D Quirke P. Identifying Stage III Colorectal Cancer Patients: The Influence of the Patient, Surgeon, and Pathologist. J Clin Oncol (2007) 25(18):2573–9. doi: 10.1200/JCO.2007.11.0445 17577036

[19] Nash G Row D Weiss A Shia J Guillem J Paty P. A Predictive Model for Lymph Node Yield in Colon Cancer Resection Specimens. Ann Surg (2011) 253(2):318–22. doi: 10.1097/SLA.0b013e318204e637 21169808

[20] Stracci F Bianconi F Leite S Liso A La Rosa F Lancellotta V. Linking Surgical Specimen Length and Examined Lymph Nodes in Colorectal Cancer Patients. Eur J Surg Oncol (2016) 42(2):260–5. doi: 10.1016/j.ejso.2015.11.017 26723169

[21] Morcos B Baker B Masri Al M Haddad H Hashem S. Lymph Node Yield in Rectal Cancer Surgery: Effect of Preoperative Chemoradiotherapy. Eur J Surg Oncol (2010) 36(4):345–9. doi: 10.1016/j.ejso.2009.12.006 20071133

[22] Goldstein M Mitchell E. Carcinoembryonic Antigen in the Staging and Follow-Up of Patients With Colorectal Cancer. Cancer Invest (2005) 23(4):338–51. doi: 10.1081/CNV-58878 16100946

[23] Beauchemin N Arabzadeh A. Carcinoembryonic Antigen-Related Cell Adhesion Molecules (CEACAMs) in Cancer Progression and Metastasis. Cancer metastasis Rev (2013) 32:643–71. doi: 10.1007/s10555-013-9444-6 23903773

[24] Hammarström S. The Carcinoembryonic Antigen (CEA) Family: Structures, Suggested Functions and Expression in Normal and Malignant Tissues. Semin Cancer Biol (1999) 9(2):67–81. doi: 10.1006/scbi.1998.0119 10202129

[25] Raoof M Nelson R Nfonsam V Warneke J Krouse R. Prognostic Significance of Lymph Node Yield in Ypn0 Rectal Cancer. Br J Surg (2016) 103(12):1731–7. doi: 10.1002/bjs.10218 27507796

[26] Gao P Song Y Yang Y Zhao S Sun Y Sun J. What Is the Minimum Number of Examined Lymph Nodes After Neoadjuvant Therapy in Rectal Cancer? J gastrointestinal Surg (2018) 22(6):1068–76. doi: 10.1007/s11605-018-3717-x 29468556

[27] Guadagni S Fiorentini G Mambrini A Masedu F Valenti M Mackay A. Multidisciplinary Palliation for Unresectable Recurrent Rectal Cancer: Hypoxic Pelvic Perfusion With Mitomycin C and Oxaliplatin in Patients Progressing After Systemic Chemotherapy and Radiotherapy, a Retrospective Cohort Study. Oncotarget (2019) 10(39):1–13. doi: 10.18632/oncotarget.26972 PMC6570475 31231460

[28] Karantanas A Yarmenitis S Papanikolaou N Gourtsoyiannis N. Preoperative Imaging Staging of Rectal Cancer. Digestive Dis (Basel Switzerland) (2007) 25(1):20–32. doi: 10.1159/000099167 17384505

[29] Leonard D Remue C Orabi Abbes N Maanen van A Danse E Dragean A. Lymph Node Ratio and Surgical Quality are Strong Prognostic Factors of Rectal Cancer: Results From a Single Referral Centre. Colorectal Dis (2016) 18(6):O175–84. doi: 10.1111/codi.13362 27128602

[30] Zhang H Hu H Guan Z Chen R Xu C Huang R. Construction of a Prognostic Nomogram for Colorectal Cancer Patients With Fewer Than Twelve Lymph Nodes Examined: A Population-Based Study in the SEER Database and China. J gastrointestinal Surg (2022) 26(1):214–7. doi: 10.1007/s11605-021-05060-8 34159554

